# Correction: Jamal et al. Preparation of 6-Mercaptopurine Loaded Liposomal Formulation for Enhanced Cytotoxic Response in Cancer Cells. *Nanomaterials* 2022, *12*, 4029

**DOI:** 10.3390/nano14050443

**Published:** 2024-02-28

**Authors:** Alam Jamal, Amer H. Asseri, Ehab M. M. Ali, Afnan H. El-Gowily, Mohamed Imran Khan, Salman Hosawi, Reem Alsolami, Tarek A. Ahmed

**Affiliations:** 1 Department of Biochemistry, Faculty of Science, King Abdulaziz University, Jeddah 21589, Saudi Arabia; ajamal0015@stu.kau.edu.sa (A.J.); ahasseri@kau.edu.sa (A.H.A.); shosawi@kau.edu.sa (S.H.); 2 Centre for Artificial Intelligence in Precision Medicines, King Abdulaziz University, Jeddah 21589, Saudi Arabia; ramalsolami@kau.edu.sa; 3 Division of Biochemistry, Department of Chemistry, Faculty of Science, Tanta University, Tanta 31527, Egypt; afnan.hamdy@science.tanta.edu.eg; 4 Department of Medical Laboratory Sciences, Faculty of Applied Medical Sciences, King Abdulaziz University, Jeddah 21589, Saudi Arabia; 5 Department of Pharmaceutics, Faculty of Pharmacy, King Abdulaziz University, Jeddah 21589, Saudi Arabia

## Error in Figure

In the original publication [1], there was a mistake in Figures 2 and 3, as published. The confusion occurred during the preparation of the Figures, leading to a mix-up between the control sample and one of the studied samples (Figure 2A,D), and the control sample and one of the studied samples (Figure 3A). The corrected Figure 2 and Figure 3 appear below.

## Text Correction

Following the error in Figure 3, there was an error in the original text description. A correction has been made to Section 3. Results and Discussion, 

3.4. Cell Cycle Analysis of HepG2 Treated with Free 6-MP and Liposomal Formulation (F1), Paragraph 1:

“When compared to untreated HepG2 cells, which were arrested in sub-G1 (5.2%) phase, G0/G1 phase (29.3%), S phase (18.6%), and G2/M (46.3%), respectively, HepG2 cells treated with 6-MP at a dose of 30 µg/mL showed an increase in sub-G1 (6.7%), G0/G1 phase (37.6%) and in S phase (21.8%) and decreased in G2/M (32.8%) (Figures 3A,B and 4)”.

The authors state that the scientific conclusions are unaffected. This correction was approved by the Academic Editor. The original publication has also been updated.

## Figures and Tables

**Figure 2:**
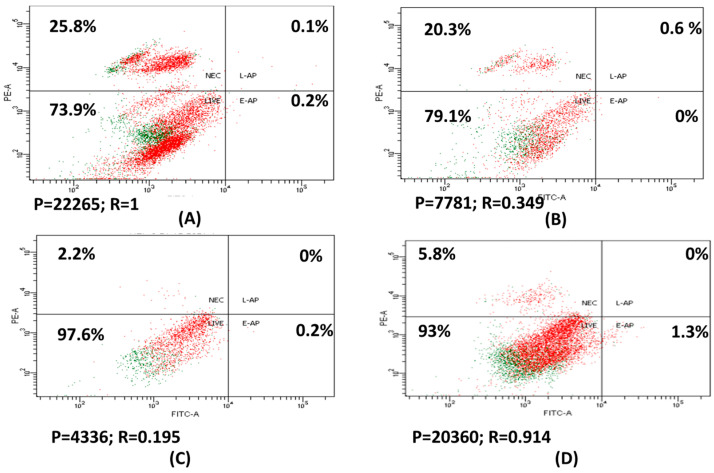
HepG2 staining with Annexin V/7-PI; control (**A**); treated with 30 µg/mL 6-MP (**B**); 5 µg/mL 6-MP loaded with positive charge liposome [F1] (**C**); and free positive charge liposomes [F3] (**D**).

**Figure 3:**
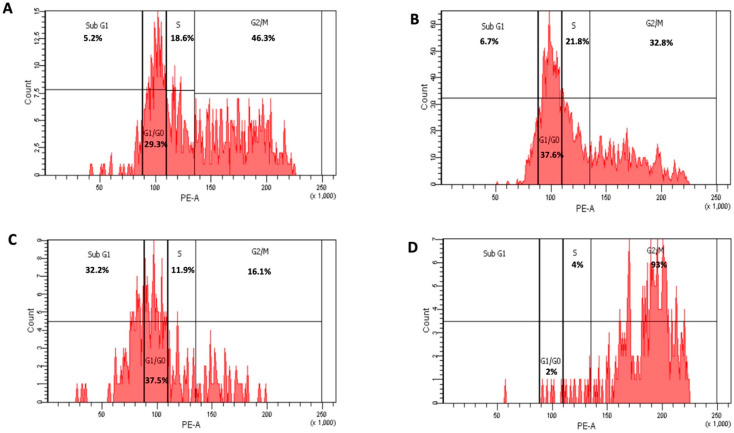
Cycle arrest of untreated HepG2, control (**A**); treated with 30 µg/mL 6-MP) (**B**); 5 µg/mL of 6-MP loaded positive charge liposomes [F1] (**C**); and drug free positive charge liposomes [F3] (**D**).

## References

[1] Jamal A., Asseri A.H., Ali E.M.M., El-Gowily A.H., Khan M.I., Hosawi S., Alsolami R., Ahmed T.A. (2022). Preparation of 6-Mercaptopurine Loaded Liposomal Formulation for Enhanced Cytotoxic Response in Cancer Cells. Nanomaterials.

